# *Inonotus obliquus* Polysaccharide Ameliorates Azoxymethane/Dextran Sulfate Sodium-Induced Colitis-Associated Cancer in Mice via Activation of the NLRP3 Inflammasome

**DOI:** 10.3389/fphar.2020.621835

**Published:** 2021-02-02

**Authors:** Jiawei Li, Chao Qu, Fangfang Li, Yifang Chen, Jinjuan Zheng, Yao Xiao, Quanxin Jin, Guihua Jin, Xuezhu Huang, Dan Jin

**Affiliations:** ^1^ Immunology and Pathogenic Biology Key Laboratory of Jilin Province, Yanbian University, Yanji, China; ^2^ Department of Anesthesiology, Affiliated Hospital of Yanbian University, Yanji, China

**Keywords:** inonotus obliquus polysaccharide, CAC, AOM/DSS, NLRP3 inflammasome, proinflammatory mediators

## Abstract

*Inonotus obliquus* polysaccharide (IOP), the primary constituent of the parasitic fungus *Inonotus obliquus*, has anti-tumor, anti-inflammatory, anti-oxidation effects. However, the roles of IOP on colitis-associated cancer (CAC) are still unclear. Herein, we tested the efficacy of IOP using a mouse model of CAC induced by azoxymethane and dextran sulfate sodium (AOM/DSS). We confirmed that intragastric administration of IOP decreased CAC-induced body weight loss, colon tissue damage, colon shortening, and expression of proinflammatory mediators. Meanwhile, IOP treatment increased in expression of the NLRP3 inflammasome, IL-1β, and IL-18 in the colon of CAC mice. Moreover, *in vitro*, IOP inhibited the proliferation of SW620 colorectal cancer cells. Finally, overexpression of NLRP3 with plasmid transfection could further enhance the activation of NLRP3 inflammasome by IOP. Taken together, these results suggest that IOP suppresses the development of CAC, possibly by activation of the NLRP3 inflammasome, and reveal that IOP may be a therapeutic drug candidate for CAC.

## Introduction

Colorectal cancer is one of the leading causes of cancer death worldwide (Saif et al., 2006; Chen et al., 2016). Colitis-associated cancer (CAC) frequently develops in patients with long-term intestinal inflammation, for example, those with inflammatory bowel disease (IBD), and tumors may also form at other sites in the body. CAC is a leading complication of IBD, and its incidence and mortality rates have increased steadily (Leong et al., 2004). Nonetheless, the link between chronic inflammation and cancer, including various theories about the progression of IBD to CAC, is not well understood. Therefore, we focused on the potential mechanism of NLRP3 inflammasomes in the process of connecting IBD and CAC.

Inflammasomes are intracellular multiprotein complexes that detect and respond to pathogenic organisms and endogenous stressors via accelerating the expression of IL-1β and IL-18. Although there are many inflammasomes, the most widely studied is the NLRP3 inflammasome (Hirota et al., 2011), consists of NLRP3, ASC, and caspase-1 (Ding et al., 2017). NLRP3 triggers innate immunity by activating caspase-1, then cleaves the inflammatory molecule IL-1β and IL-18, which induces inflammation and eliminate tumor cells (Dai et al., 2020). Indeed, NLRP3 inflammasome plays a key regulatory role in inflammation-induced cancer. Zhao et al. found that GL-V9, a small molecule activator of 5′ adenosine monophosphate-activated protein kinase, can decrease AOM/DSS-induced CAC by activating the NLRP3 inflammasome (Zhao et al., 2017). Chung et al. demonstrated that infection with *Enterococcus faecalis* could modulate activation of the NLRP3 inflammasome and inhibit CAC development (Chung et al., 2019). Therefore, a better understanding of inflammasomes will be crucial for the treatment for CAC diseases.

*Inonotus obliquus* (Ungerbäck et al., 2012) is a parasitic tree fungus that contains several bioactive components, including polysaccharides, lignin, embolic acid, and melanin. Among these, *Inonotus obliquus* polysaccharides (IOP) have documented anti-tumor (Jiang et al., 2020), anti-inflammatory (Hu et al., 2017), and anti-oxidation (Sim et al., 2016) effects, leading to increased interest in their potential clinical applications. Fan et al. found that IOP inhibits the progression of gastric cancer by stimulating lymphocyte activity (Fan et al., 2012), while Lee et al. illustrated that IOP inhibits the invasion and migration of B16-F10 melanoma cells (Lee et al., 2014). Ning et al. demonstrated that IOP has an inhibitory effect on the proliferation of cultured U251 human glioma cells (Ning et al., 2014). In addition, our previous work showed that IOP can inhibit the development of DSS-induced colitis in mice (Chen et al., 2019). In this study, we employed the AOM/DSS-induced mouse model of CAC to explore the effects and potential mechanisms of action of IOP in CAC.

## Materials and Methods

### Reagents

Main reagents were obtained as follows: AOM (Sigma-Aldrich, St. Louis, MO, USA); DSS (MP, Solon, OH, USA); DMEM and FBS (Biological Industries, BeitHaEmek, Israel); CCK-8 kit (Beyotime, Jiangsu, China); ELISA kits for human IL-18 and IL-1β (Mei Biao Biological, Shanghai, China); MCC950 (MedChemExpress, Monmouth Junction, NJ, USA); Lipofectamine 2000 transfection reagent (Invitrogen, Carlsbad, CA, USA); P3*flag-NLRP3 (Public Protein/Plasmid Library, Shanghai, China); ECL detection reagent (Everbright, San Ramon, CA, USA); anti-NLRP3, anti-caspase-1, anti-IL-18 (all from Abcam, Cambridge, UK); anti-ASC, anti-IL-1β, anti-IL-6, anti-COX-2, anti-TNF-α, and anti-β-actin (all from Cell Signaling Technology, Boston, MA, USA); HRP-conjugated goat anti-rabbit and anti-mouse IgG (Alfetronic, Beijing, China).

### Preparation of *Inonotus obliquus* polysaccharide

IOP was obtained from the Department of Immunology and Pathogenic Biology of Yanbian University. The preparation of IOP was described as previously (Chen et al., 2019).

### Animal Experiments

Male BALB/c mice (six to eight weeks of age, weighing 20 ± 2 g) were supplied via Evans Laboratory Animal Technology and allowed to acclimate for one week of adaptive feeding. The mice were classified into three groups. Starting on day 0, each group was orally administered 0.9% sterile saline (0.3 ml) once daily and provided standard chow for 10 days. The control group (n = 5) continued on standard chow from day 11 to day 101. On day 11, the model group were given a single injection of AOM (10 mg/kg) (AOM/DSS + saline; n = 12). On day 17, mice were given 2.5% DSS (w/v) in drinking water for seven consecutive days, and then given fresh drinking water for 14 days (Tanaka et al., 2003; Yoshida et al., 2007). 7 days of DSS and 14 days of fresh water as a cycle, repeated 4 times until day 101. The experimental group (AOM/DSS + IOP; n = 12) was treated as for the model group except IOP was gavaged (150 mg/kg in water) every other day from day 0 to day 101 days. The study followed the Yanbian University ethical guidelines for animal care and use.

### Evaluation of Colitis-Associated Cancer in Mice

Mice were observed and weighed daily until sacrifice. The criteria for evaluating CAC included body weight, stool consistency, and anal prolapse. On day 101, mice were sacrificed, the colon was collected, and macroscopic tumors were measured and counted with digital calipers under a dissecting, and colon adenomas were weighed.

### Cell Culture

The SW620, a human colon cancer cell line, was purchased from the American Type Culture Collection. The cells were cultured in DMEM medium containing 10% FBS, 1% penicillin-streptomycin in an incubator with 5% CO2 at 37°C.

### Cell Proliferation Assay

SW620 cells (1 × 10^4^ cells/well) were inoculated in 96-well plates and cultured with various concentrations of IOP. At the indicated time, 10μL CCK-8 was added to each well, incubated for 2 h. The absorbance was measured at 450nm.

### Migration Assay

SW620 cells (2 × 10^6^ cells/well) were plated in 6-well plates. After the cells had attached to the wells, a wound was lacerated through the cell layer with a sterile pipette dripper, the non-adherent cells were rinsed off. The cells were cultured with IOP for 48 h and photographed for 0 h and 48 h. Then the wound size and migration distance were calculated.

### Colony-Forming Assay

SW620 cells (500 cells/well) were seeded into 6-well plates and treatment with IOP for 48 h. The medium was then exchanged for medium without IOP and the cells were cultured for 1 week. The cells were stained with Giemsa solution (Solarbio) for 15 min, photographed, and colonies were counted.

### Histological Analysis

The tissue were paraffin-embedded, sectioned (thickness 4 l m), and stained with H&E. The images were observed using a microscope, and performed histological analysis. Standard diagnosis as described by Robertis (Robertis et al., 2011), and the scoring criteria are listed in Table 1.

**TABLE 1:** Histological scoring standard.

Score	Mucosal architecture	Reactive epithelial hyperplasia	Inflammatory cell infiltrate
0	Normal	Normal	None
1	Irregular crypts	Mildhyperplasia, minimalgoblet cellloss	Low level of inflammation with scattered infiltrating, local lesion
	Non-parallel crypts		
2	Bifurcation and branched crypts	Moderate hyperplasia, mild goblet cell loss	Moderate inflammation with multipe foci
3	Crypt loss	Marked hyperplasia with moderate to marked goblet cell loss	High level of inflammation with marked wall thickening

### Real-Time Quantitative PCR

From tissues and cells, the total RNA isolation was executed using Trizol, which was then reverse-transcribed with Oligo-dt primers, and the amplification were carried out using a Stratagene Mx3005P PCR system. The results are expressed as the ratio of the mRNA of interest to that of β-actin or GAPDH. The relevant primer sequences are listed in Table 2.

**TABLE 2:** Primer sequences for RT-qPCR.

Gene	Source	Forward sequence	Reverse sequence
NLRP3	Mouse	GAG​TTC​TTC​GCT​GCT​ATG​T	ACCTTCACGTCTCGGTTC
Caspase-1	Mouse	TAT​CCA​GGA​GGG​AAT​ATG​TG	ACA​ACA​CCA​CTC​CTT​GTT​TC
ASC	Mouse	ACA​CTT​TGT​GGA​CCA​GCA​CA	CAC​GAA​CTG​CCT​GGT​ACT​GT
IL-18	Mouse	AGT​AAG​AGG​ACT​GGC​TGT​GAC​C	TTG​GCA​AGC​AAG​AAA​GTG​TC
IL-1β	Mouse	CAA​CCA​ACA​AGT​GAT​AAT​TCT​CG	GAT​CCA​CAC​TCT​CCA​GCT​GCA
TNF-α	Mouse	GGC​AGG​TCT​ACT​TTG​GAG​TCT​G	ACA​TTC​GAG​GCT​CCA​GTC​ATC​G
IL-6	Mouse	CCA​CTG​GTG​GAC​CAG​CTC​A	CTC​GCA​CTG​ACT​GTT​ACT​AT
β-actin	Mouse	TCT​GGT​CGT​ACC​ACA​GGC​AT	CGC​TCG​TTG​CCA​ATA​GTG​AT
NLRP3	Human	TGG​CTG​TAA​CAT​TCG​GAG​ATT​G	GAA​GTC​ACC​GAG​GGC​GTT​GT
Caspase-1	Human	GGA​AAC​AAA​AGT​CGG​CAG​AG	ACG​CTG​TAC​CCC​AGA​TTT​TG
ASC	Human	GGT​CAC​AAA​CGT​TGA​GTG​GC	AGA​GCT​TCC​GCA​TCT​TGC​TT
IL-18	Human	TGA​CCA​AGG​AAA​TCG​GCC​TC	GAG​GAT​TGG​GAC​TAG​GCA​CGG
IL-1β	Human	AAT​CAA​ATT​TTG​CCG​CCT​CG	CGT​GCA​GTT​CAG​TGA​TCG​TA
GAPDH	Human	CCA​CAT​CGC​TCA​GAC​ACC​AT	GCA​ACA​ATA​TAC​CAC​TTT​ACC​A

### Immunochemistry

Samples of mouse colon tissue were paraffin-embedded and sectioned. The sections were incubated with primary antibody ASC, caspase-1, NLRP3, IL-18, IL-1β, or COX-2 at 4°C for 12 h, then secondary antibodies were added and incubated for 30 min. After washing, color was developed by addition of DAB, and counter-stained with hematoxylin. Finally, a microscope was used for examination.

### Western Blot

Tissues and cells were lyzed in RIPA lysis buffer. The proteins were isolated on 8%–15% SDS-PAGE gels (VWR, Jiangsu, China), then transferred to PVDF membranes. The imprints were incubated with rabbit primary antibodies against human or mouse NLRP3, ASC, caspase-1, IL-18, IL-1β, COX-2, TNF-α, IL-6, or β-actin for 12 h. The blots were washed, incubated with secondary antibodies for 1 h. Finally, the ECL reagent was used for detection, and the blots were developed and imaged.

### ELISA

Concentrations of IL-1β and IL-18 in the supernatants of cultured SW620 cells were measured using ELISA kits.

### Statistical Analysis

All experiments were repeated three times, and the data are presented as the mean ± SD. Group means were compared using a *t* test. *p* < 0.05 was considered statistically significant.

## Results

### Effects of *Inonotus obliquus* Polysaccharide in a Mouse Model of Azoxymethane/Dextran Sulfate Sodium-Induced Colitis-Associated Cancer

**FIGURE 1:**
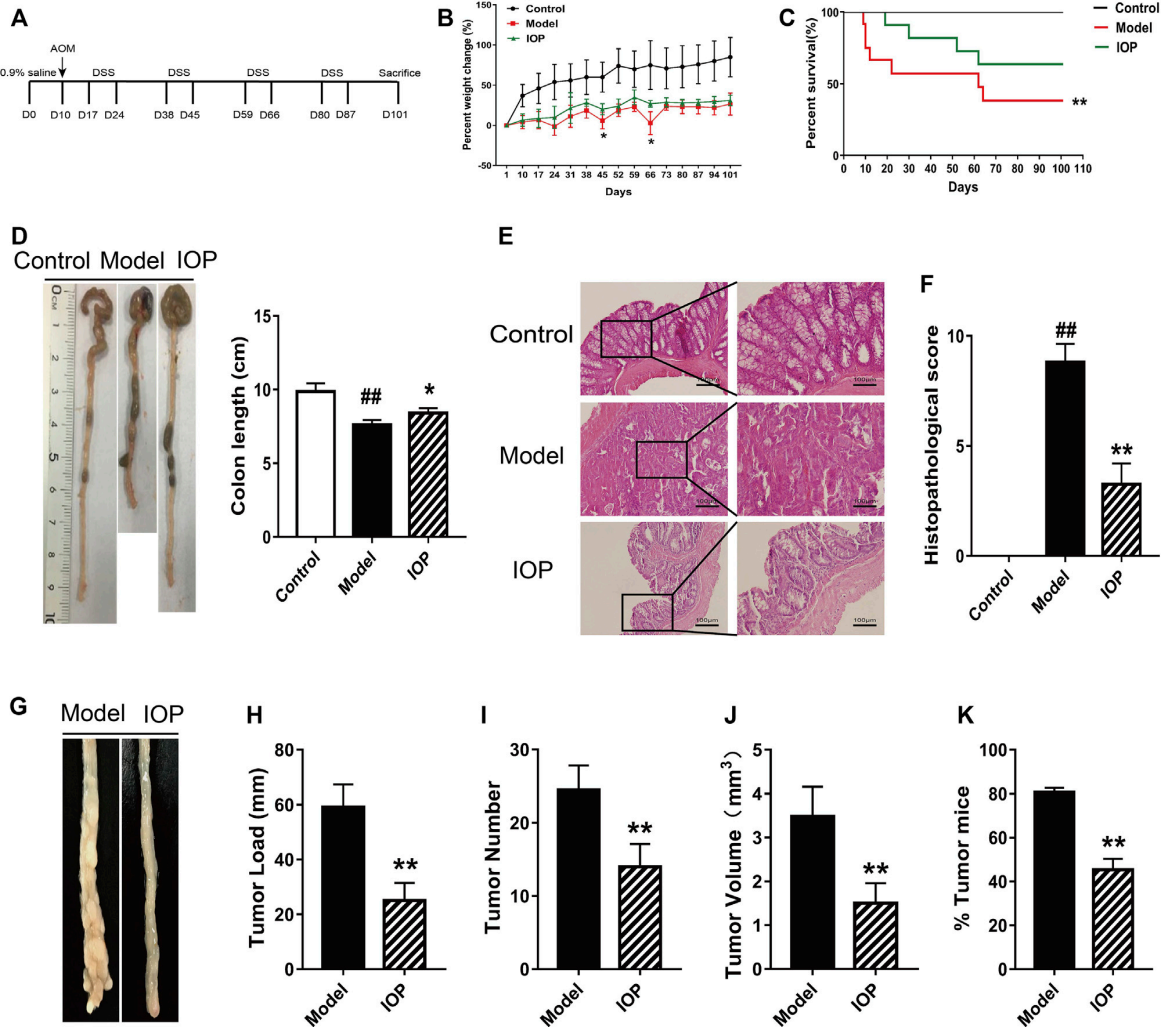
IOP treatment reduces the severity of AOM/DSS-induced CAC in mice. **(A)** A schematic of the CAC model. **(B)** Body weight change of mice. **(C)** Survival of mice after 101 days. **(D)** Colon lengths. **(E–F)**, Representative images of colon histopathology, and semiquantitative histological score (original magnification ×200 or ×400, scale bars = 100 μm). **(G–J)** Total tumor numbers observed in the whole colon **(G)** tumor image **(H)** tumor load **(I)** tumor numbers **(J)** tumor volume. **(K)** Percentage of tumor in mice. Data are expressed as the mean ± SD. ##*p* < 0.01 vs the control group; *p < 0.05, **p < 0.01 vs the model group.

To evaluate whether IOP can mitigate or prevent CAC, we administered AOM/DSS to two groups of BALB/c mice and treated them with vehicle (model group, n = 12) or IOP (150 mg/kg) every other day for 101 days (IOP group, n = 12). A third group of mice were untreated (no AOM, DSS, or IOP) and served as the control group (n = 5) (Figure 1A). After 101 days, IOP group revealed more remission symptoms, including less loss of body weight, higher survival rate, longer colon length than model group (Figures 1B–D). Meanwhile, histological examination exhibited neoplastic glands, colonic epithelial cell disorder as well as larger cell nuclei in the model group, whereas these effects were markedly ameliorated by IOP treatment (Figures 1E,F). Furthermore, tumors developed in the colons of both the model and IOP group, but tumor burdens were markedly lost in IOP group over model group (Figures 1G,H). And, statistically significant differences in tumor number and tumor volume could be observed (Figures 1I,J). Notably, the number of tumors in the colon tissue of mice enhanced from 45% in the IOP group to 80% in the model group (Figure 1K). These results verified that IOP reduced CAC severity in mice induced by AOM/DSS.

### *Inonotus obliquus* Polysaccharide Decreases Colonic Expression of Inflammatory Mediators in Mice With AOM/DSS-Induced CAC

**FIGURE 2:**
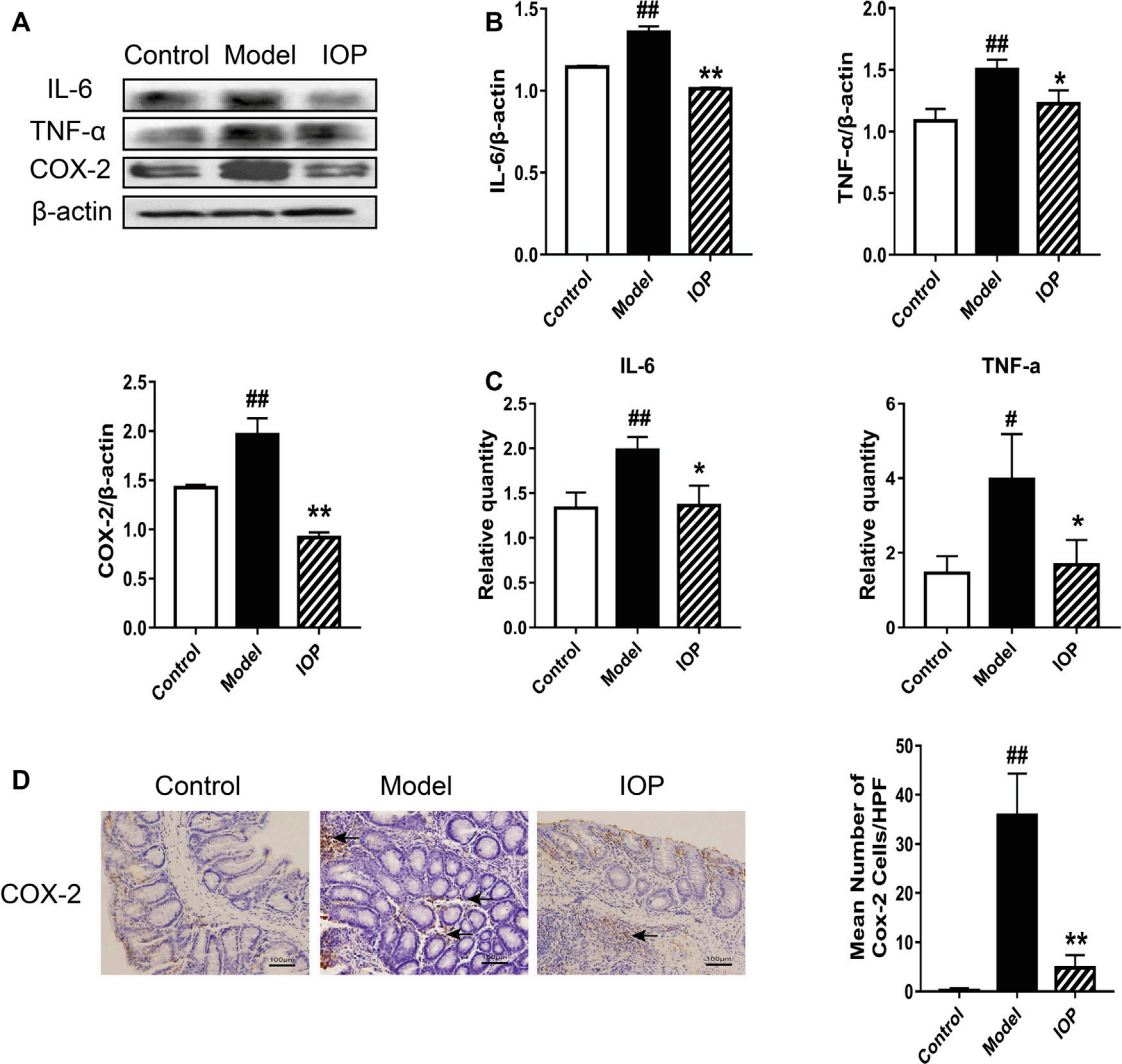
IOP decreases the expression of inflammatory mediators in the colons of mice with AOM/DSS-induced CAC. **(A–B)** The levels of IL-6, TNF-α, COX-2 in colon tissues was monitored using Western blot. **(C)** Expression of IL-6, TNF-α, COX-2 in colon tissues was detected using Real-time PCR. **(D)** The collected colon tissue sections were subjected to COX-2 immunostaining. Black arrows indicate COX-2 positive cells (original magnification ×400, scale bars = 100 μm). Data are expressed as the mean ± SD., #*p* < 0.05, ##*p* < 0.01 vs the control group; *p < 0.05, **p < 0.01 vs the model group.

It is known that the activation of cyclooxygenase 2 (COX-2) catalyzes the production of certain inflammatory mediators and can enhance the progression of colitis to CAC; indeed, increased production of COX-2 and other inflammatory mediators is a hallmark of CAC. Thus, we explored the role of IOP on the levels of COX-2 and related cytokines in the colons of mice with AOM/DSS-induced CAC. Western Blot analysis revealed that IL-6, TNF-α, and COX-2 protein expressions were also decreased by IOP treatment (Figures 2A,B) and, similarly, the gene expression of proinflammatory cytokines (IL-6 and TNF-α) in the colons of the IOP group was dramatically lower than that of the model group (Figure 2C). IHC staining of COX-2 in colon sections confirmed these results (Figure 2D). Thus, IOP decreases the expression of inflammatory mediators during AOM/DSS-induced CAC.

### *Inonotus obliquus* Polysaccharide Promotes Activation of the NLRP3 Inflammasome in the Colons of Mice With AOM/DSS-Induced CAC

**FIGURE 3:**
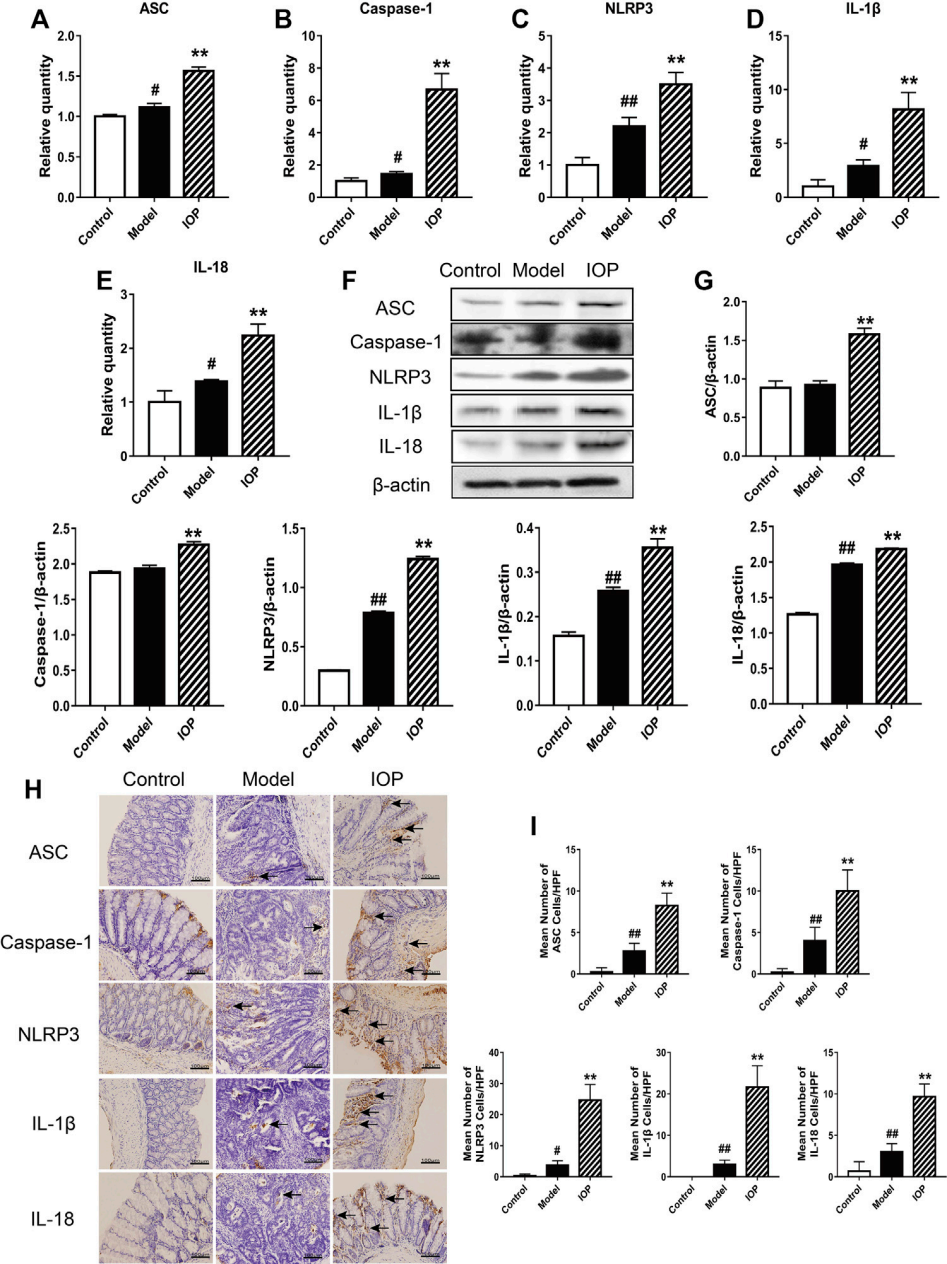
IOP promotes activation of the NLRP3 inflammasome in mice with AOM/DSS-induced CAC. **(A–E)** Expression of NLRP3 inflammasome **(A–C)**, IL-1β **(D)** and IL-18 **(E)** in colon tissues was examined using Real-time PCR. **(F–G)**, The levels of ASC, caspase-1, NLRP3, IL-1β, IL-18 in colon tissues was detected by Western blot. **(H–I)**, The collected colon tissue sections were performed to NLRP3 inflammasome, IL-1β and IL-18 immunostaining. Black arrows indicate positive cells of the associated protein (original magnification ×400, scale bars = 100 μm). Data are expressed as the mean ± SD. #*p* < 0.05, ##*p* < 0.01 vs the control group; *p < 0.05, **p < 0.01 vs the model group.

The NLRP3 inflammasome has been reported to play an essential role in the AOM/DSS-induced CAC process in mice. Therefore, we explored whether IOP affects the expression and/or activity of the NLRP3 inflammasome in colon tissues. The expressions of NLRP3 inflammasome, IL-1β and IL-18 were evaluated via western blot and real-time PCR. These results indicated increased expression of the inflammatory components ASC, caspase-1 and NLRP3 and downstream cytokines IL-1β and IL-18 in the model group in comparison to the control group (Figures 3A–G), indicating that NLRP3 inflammasome was up-regulated during CAC development. And, their expressions were further enhanced by IOP treatment (Figures 3A–G), suggesting that NLRP3 inflammasome may both contribute to protection against tumorigenesis. Moreover, immunohistochemical analysis indicated colons of IOP group contained significantly more cells staining positive for NLRP3 inflammasome, IL-1β and IL-18 (Figures 3H,I). Taken together, these results demonstrated that IOP can promote the activation of NLRP3 inflammasomes induced by AOM/DSS, thus providing a potential mechanism for studying the benefits of IOP in the development of CAC.

### *Inonotus obliquus* Polysaccharide Inhibits the Growth of SW620 Cells *in vitro*

**FIGURE 4:**
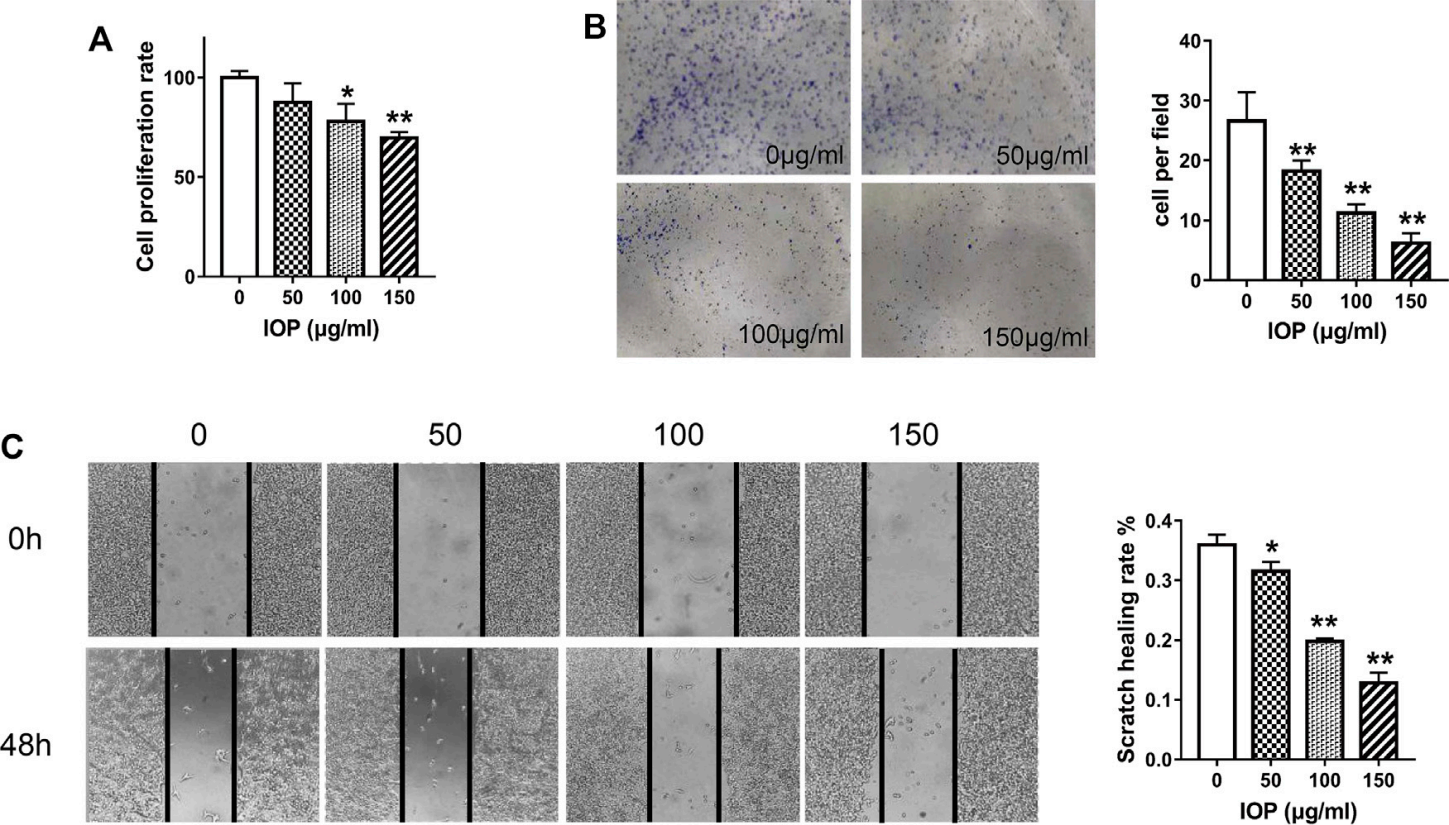
IOP inhibits the tumorigenic behavior of SW620 cells *in vitro*. **(A)** CCK-8 proliferation assay of SW620 cells after 48 h of IOP treatment. **(B)** Colony formation by SW620 cells after 1 week of IOP treatment. **(C)** Wound healing migration assay of SW620 cells after 48 h of IOP treatment. *p < 0.05, **p < 0.001 vs the control group.

To investigate the anti-tumor effects of IOP directly, we inspected growth of the human colon cancer cell line (SW620) *in vitro*. The cells were treated with IOP for 48 h and determined by CCK-8 assay. As shown in Figure 4A, IOP availably down-regulated the growth of SW620 cell. Simultaneously, we evaluated the effect of IOP on the colony-forming ability of SW620 cells, and found that IOP treatment dose-dependently reduced the number of colonies formed after 7 days in culture (Figure 4B). Moreover, IOP treatment also inhibited the migration of SW620 cells in a wound healing assay, particularly at high IOP concentrations (Figure 4C). These results indicated that IOP suppressed the growth of SW620 cells *in vitro*.

### *Inonotus obliquus* Polysaccharide Increases NLRP3 Inflammasome Signaling in SW620 Cells

**FIGURE 5:**
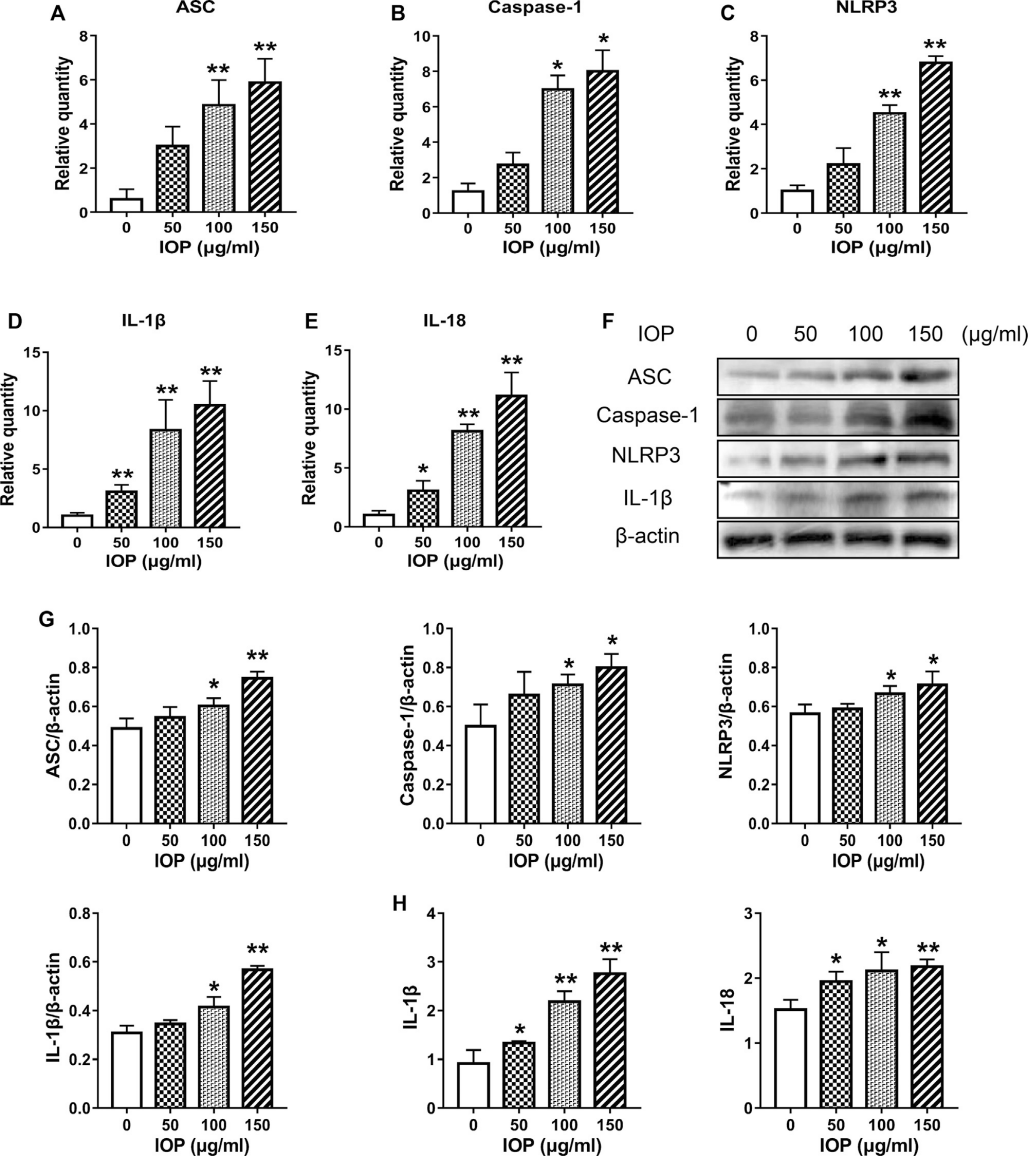
IOP increases NLRP3 inflammasome signaling in SW620 cells. **(A–E)**, Real-time PCR was used to inspect the levels of ASC **(A)**, caspase-1 **(B)**, NLRP3 **(C)**, IL-1β **(D)**, and IL-18 **(E)** in SW620 cells. F-G, The cells were collected and the expression of ASC, caspase-1, NLRP3 and IL-1β was examined by Western blot. H, Expression of IL-1β and IL-18 in the supernatant of SW620 cells treated with IOP was analyzed by ELISA. Data are presented as the mean ± SD. *p < 0.05, **p < 0.01 vs the control group.

IOP has been shown to activate the NLRP3 inflammasome and promote the production of cytokines in the colon of CAC mice (Figure 3), and we further verified whether IOP has the same effect on SW620 cells *in vitro*. As shown in Figure 5, we found that IOP treatment significantly enhanced the expression of NLRP3 inflammasome at the mRNA levels (Figures 5A–E). Furthermore, we detected the expression of NLRP3 inflammasome activation related proteins, and the results verified that IOP observably up-regulated the levels of ASC, caspase-1, NLRP3 proteins *in vitro* (Figures 5F,G). As expected, IOP also enhanced the secretion of cytokines IL-1β and IL-18 in tumor cells (Figures 5D,E,H).

### The Anti-tumor Activity of *Inonotus obliquus* Polysaccharide Is Mediated by Activation of the NLRP3 Inflammasome

**FIGURE 6:**
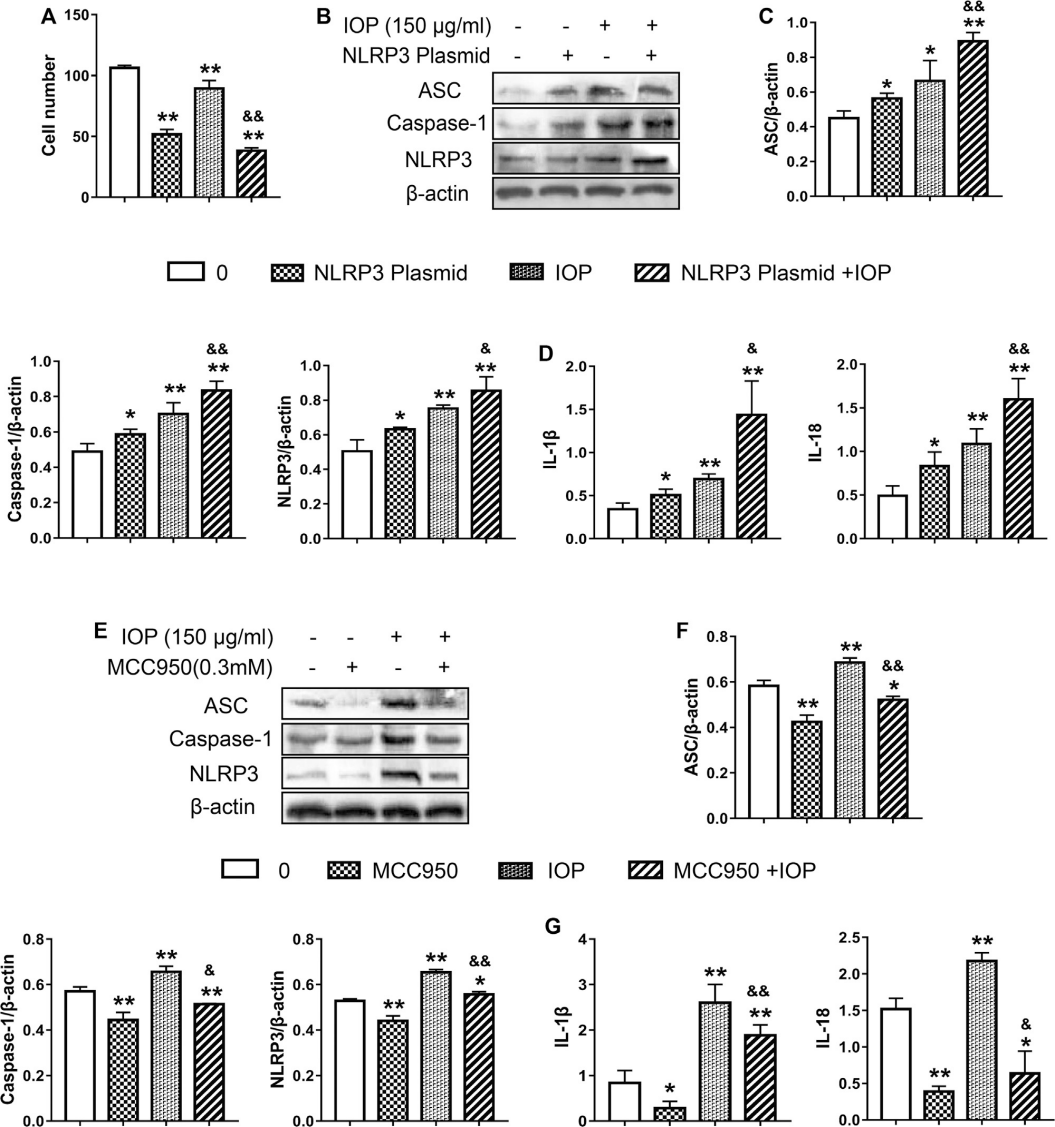
The anti-tumor activity of IOP is mediated by promotion of NLRP3 inflammasome activation. **(A–D)**, SW620 cells were transfected with NLRP3 plasmid for 6 h. Then transfected SW620 cells were treated with IOP (150 ug/ml) alone for 48 h **(A)** Cell proliferation assay **(B and C)** Expression of NLRP3 inflammasome were measured by Western blot **(D)** The production of IL-1β and IL-18 in the cell supernatant was analyzed via ELISA. E-G, SW620 cells were stimulated for 60 min with NLRP3 inhibitor (MCC950, 0.3mM), and then cultured with IOP (150g/ml) for 48 h **(E and F)** The protein of NLRP3 inflammasome in SW620 cell lysate was examined using Western blot **(G)** IL-1β and IL-18 secretion measured by ELISA. Data are expressed as the mean ± SD. *p < 0.05, **p < 0.01 vs the control group; ^&^ *p* < 0.05, ^&&^ *p* < 0.01 for the NLRP3-transfected group vs the NLRP3/IOP group and for the MCC950-treated group vs the MCC950/IOP group.

To determine whether IOP activation of the NLRP3 inflammasome is causally related to its anti-tumor activity, we examined the effects of NLRP3 inflammasome inhibition (using a small molecule inhibitor MCC950) or activation (transfected NLRP3 overexpressed plasmid) on the growth of SW620 cell. After transfection with NLRP3 plasmid, overexpression of NLRP3 alone was observed to inhibit cell proliferation. Moreover, IOP further increased the inhibitory effect of NLRP3 plasmid on the growth of SW620 cells (Figure 6A). As expected, the NLRP3 plasmid efficiently increased the NLRP3 protein (Figure 6B). Likewise, NLRP3 overexpression significantly increased IOP-induced NLRP3 inflammasome activation and cytokine production compared with either IOP treatment or NLRP3 plasmid transfection alone (Figures 6B–D). In contrast, treatment with the NLRP3 suppressed IOP-mediated activation of NLRP3 inflammasome activity and cytokine production (Figures 6E–G). These findings above demonstrate that the anti-tumor effects of IOP are mediated, at least in part, by promoting NLRP3 inflammasome activation.

## Discussion

The mechanisms by which chronic inflammation leads to carcinogenesis are complex. In the cases of CAC associated with IBD, disease severity, duration and inflammation degree were all associated with tumor development (Larsen et al., 2016). Therefore, effective treatment of IBD plays an important role in suppressing the process of CAC. Currently, IBD is widely treated with 5-aminosalicylic acid, corticosteroids, guanidinopurine and TNF-α blockers (Alexakis et al., 2017); however, the relationship between these drugs and the development and progression of CAC is unclear. Thus, novel safe and effective drugs for CAC are urgently needed. Our study revealed an innovative approach to the treatment of CAC in mice using IOP, a valuable medicinal fungus. The study investigated the possible mechanism of AOM/DSS-induced CAC treated by IOP. The result obviously showed that IOP ameliorated the symptoms and signs of CAC induced by AOM/DSS, alleviated the pathological damage to colonic tissues, and reduced the expression level of inflammatory factors. Further studies showed that the affect of IOP might be associated with the expression of NLRP3 inflammasomes. In addition, the study revealed that IOP promotes NLRP3 inflammasome activation, thereby exerting anti-tumor effects.

TNF-α, IL-6 and COX-2 are considered to be important cytokines linking inflammation and cancer, which are also recognized to play an indispensable role in the development and progression of CAC (Triantafillidis et al., 2009). Xiao et al. confirmed that DSS induces a dramatic increase in the production of IL-6 and TNF-α in colonic tissues of mice (Xiao et al., 2016). Chen et al. also found that in the AOM/DSS model, IL-6 expression increased with the severity of the lesions (Chen et al., 2015). Here, IOP can inhibit the production of IL-6, TNF-α and COX-2 in colon tissue, suggesting that this may be one of the potential mechanisms by which IOP exerts its anti-tumor effect.

Chronic inflammation is a significant factor in the development, promotion, and progression of cancer (Nathan et al., 2010). NLRP3 plays a vital role in inflammation, and activation of NLRP3 inflammasome is associated with inflammation-induced cancer. However, the impact of NLRP3 inflammasome in IBD and CAC is controversial. Chen et al. demonstrated that inflammasome activity could promote the occurrence of inflammatory diseases and CAC via regulation of intestinal homeostasis and anti-microbial immunity (Chen et al., 2011). Bauer et al. also indicated that activation of NLRP3 significantly enhanced the risk of IBD (Bauer et al., 2010). However, in stark contrast, other studies has demonstrated that the NLRP3 inflammasome have a protective effect in colitis (Zaki et al., 2010). Allen et al. indicated that the size of colon tumors in AOM/DSS-treated mice deficient in NLRP3, caspase-1, and ASC was larger than that seen in similarly treated wild-type mice (Allen et al., 2010). Zaki et al. found that NLRP3 activation inhibits AOM/DSS-induced CAC (Zaki et al., 2010), supporting an affect for the NLRP3 inflammasome in preventing the occurrence of CAC. Interestingly, we found that the IOP increased the expression of NLRP3 inflammasome in colon tissues and upregulated the production of IL-1β and IL-18. It indicated that IOP prevented mice from AOM/DSS-induced CAC via activating NLRP3 inflammasome.

We confirmed and extended our *in vivo* observations by examining the anti-tumor effects of IOP on SW620 cells *in vitro*. Our results confirmed that IOP inhibited the growth, migration and colony-forming ability of SW620 cells. We also found that IOP up-regulated the level of NLRP3 inflammasome and promoting NLRP3 inflammasome activation. And, IOP could increase the generation of IL-1β and IL-18. To further prove these findings, we transfected SW620 cells with NLRP3 plasmid. After transfection with NLRP3 plasmid, NLRP3 overexpression significantly inhibited SW620 cell proliferation and further enhanced the promotion effect of IOP on NLRP3 inflammasome. In contrast, the promotion impact of IOP on NLRP3 inflammasome activation was significantly blocked by NLRP3 inhibitors. In summary, these data supported our *in vivo* findings and verified our assumption that IOP might play an anti-tumor effect by promoting the activation of NLRP3 inflammasome.

In summary, we have demonstrated that using cell lines and mouse models, IOP as an effective treatment for CAC, and the underlying mechanism may be relevant to the activation of the NLRP3 inflammation. In combination, IOP may have utility as a potential treatment for CAC and, possibly, other tumors.

## Data Availability Statement

The original contributions presented in the study are included in the article/Supplementary Material, further inquiries can be directed to the corresponding authors.

## Ethics Statement

The animal study followed the Yanbian University ethical guidelines for animal care and use. And approved by the Animal Experiment Center Committee of Yanbian University.

## Author Contributions

DJ and XH designed this experiment. JL, CQ and FL conducted the experiments and analyzed the data. YC, JZ, YX, QJ, and GJ also participated the experiment. All authors participated in the production and modification of the manuscript.

## Funding

This work was supported by the National Natural Science Foundation of China (grant no. 81860288).

## Conflict of Interest

The authors declare that the research was conducted in the absence of any commercial or financial relationships that could be construed as a potential conflict of interest.
